# Structure and Deformation Behavior of Polyphenylene Sulfide-Based Laminates Reinforced with Carbon Fiber Tapes Activated by Cold Atmospheric Plasma

**DOI:** 10.3390/polym16010121

**Published:** 2023-12-29

**Authors:** Pavel V. Kosmachev, Sergey V. Panin, Iliya L. Panov, Svetlana A. Bochkareva

**Affiliations:** 1 Microelectronics of Multispectral Quantum Introscopy Laboratory of the R&D Center “Advanced Electronic Technologies”, National Research Tomsk State University, 634050 Tomsk, Russia; panov.iliya@mail.ru; 2 Laboratory of Mechanics of Polymer Composite Materials, Institute of Strength Physics and Materials Science of Siberian Branch of Russian Academy of Sciences, 634055 Tomsk, Russia; svp@ispms.ru (S.V.P.);

**Keywords:** carbon fiber, low-temperature atmospheric plasma, discharge with runaway electrons, PPS, laminates, reactive groups, adhesion, stress–strain state, FEM

## Abstract

Low-temperature plasma treatment with atmospheric discharge with runaway electrons (DRE) was shown to be an efficient way to activate carbon fiber’s (CF) surface and subsequently increase its interlayer shear strength (ILSS) values. It was demonstrated that an acceptable ILSS level was achieved after a DRE plasma treatment duration of 15 min. The treatment of CFs resulted in their surface roughness being increased and their functional groups grafting. The XPS data showed a change in the chemical composition and the formation of reactive oxygen-containing groups. SEM examinations of the PPS/CF laminates clearly demonstrated a difference in adhesive interaction at the PPS/CF interface. After the DRE plasma treatment, CFs were better wetted with the polymer, and the samples cohesively fractured predominantly through the matrix, but not along the PPS/CF interface, as was observed for the sample reinforced with the untreated CFs. The computer simulation results showed that raising the adhesive strength enhanced the ILSS values, but reduced resistance to transverse cracking under the loading pin. In general, higher flexural strength of the PPS/CF laminates was achieved with a greater interlayer adhesion level, which was consistent with the obtained experimental data.

## 1. Introduction

Thermoplastic polymers reinforced with high-modulus fibers are multiphase systems that attract more attention from researchers compared to thermosetting-based composites [1,2,3,4]. Among their numerous advantages are resistance to damage and cracking, ease of consolidation as well as fast and economical manufacturability.

Polyphenylene sulfide (PPS) is a common thermoplastic semicrystalline polymer that is used for a wide range of engineering applications, belonging to the class of high-performance polymers (HPPs) [5]. PPS possesses high resistance to external impacts, great mechanical properties as well as an excellent ability to maintain both shape and size even at elevated temperatures (above 200 °C) [6,7,8]. Depending on the type of applied load, PPS-based composites are able to operate at temperatures up to 240 °C (270 °C under short-term exposure). Such conditions are typical for the engine compartment of a car or electrical appliances, for example. This is the key reason for the widespread use of PPS in the automotive and electrical equipment industries. In addition, PPS is characterized by both minimal moisture absorption and great chemical resistance in relation to almost all solvents, most acids and alkalis as well as air oxygen. Other important advantages of PPS are low creep, as well as minimal permeability to most liquids and gases [9,10,11,12].

Among the polymer composites, including HPP-based ones, laminates are widespread and in demand due to their high elastic modulus and strength characteristics (along the reinforcement plane). However, they have minimal resistance to loading under both transverse rupture and interlayer shear, which can cause cracking and gradual delamination, resulting in failure. Eliminating this issue requires improving the bonding strength between their layers, which is mainly achieved by increasing interlayer adhesion. Additionally, compression sintering modes can be optimized [13,14], and some other approaches can be employed for solving this problem [15].

One of the ways to enhance resistance to interlayer cracking is a technique based on introducing an additional (‘transitional’) interlayer into laminates. They include thermoplastic and thermoset films, micro- or nanofiber non-woven mats, etc. [16,17,18,19,20]. For instance, laying carbon nanotube films (Buckypapers) increased the interlaminar fracture toughness by 45.9% for polymers reinforced with carbon fibers (CFs) [18]. Khan S. U. et al. [19] showed an increase in the interlayer shear strength (ILSS) of 31% in multiscale composites loaded with CFs. At the same time, Rojas J. A. et al. reported [20] that layering Buckypapers led to increased pore formation and poor wettability in PPS/CF laminates under incorrectly selected sintering conditions. This fault was accompanied by reduced mechanical properties, including a decrease in ILSS of ~27%, compared to those for similar samples without transition layers.

The chemical functionalization of fibers, as a way to increase interfacial adhesion in a composite, involves the use of reagents (chemicals), which can graft active chemical functional groups, or be adhered on them. Such procedures form a surface adhesion layer based on the used reagent, which improves the interaction between fibers and a matrix.

One of the most common methods for the preliminary treatment of CFs is oxidation with nitric acid that forms functional groups on their surfaces [21,22]. Zhang G. et al. showed the evolution of CFs treated with ultrasound in a H_2_SO_4_/HNO_3_ concentrated acid mixture. In addition, a number of sizing reagents can be applied [23]. Fan R. et al. [24] functionalized CFs with poly (amido amine), enhancing the interlaminar fracture shear strength (IFSS) of epoxy/CF composites by up to 85%. For the PPS/CF ones, poly (amic acid) ammonium salt can be used as a sizing agent [25], increasing the ILSS, flexural strength and elastic modulus values by 19.5%, 24.9% and 10.1%, respectively.

The thermal modification (oxidation) of CFs promotes interfacial bonding in polymer composites due to the formation of polar oxygen-containing groups on the surface of the formers [26]. The air oxidation of CFs has proven effectiveness in enhancing IFSS for composites based on both thermosetting [27] and thermoplastic [28] matrices. In [29], Chukov D. et al. reported that the heat treatment of CFs in air at 500 °C for 10 min provides them with better adhesion to PPS, but their mechanical properties sharply decreased. Thus, it cannot be implemented for manufacturing high-strength composites.

An alternative method for increasing adhesion in polymer laminates is CF surface treatment with cold plasma [30,31,32,33]. In this method, it interacts with ions, free electrons, excited atoms and molecules, as well as radicals, increasing the free energy and forming active polar groups [34,35,36]. In addition, microroughness increases on the surface of CFs upon plasma treatment, which, according to the mechanical interlocking theory [37,38,39], contributes to an additional increase in adhesion between fibers and polymers [40,41]. Considering the above advantages of the plasma treatment of CFs, many authors are focused on studying this method [42,43], in particular, when fabricating PPS/CF laminates [44,45,46,47]. This fact emphasizes the relevance of the topic.

Regardless of numerous studies on the cold atmospheric plasma modification of carbon fibers, the efficiency of enhancing interfacial strength largely depends on particular machines and parameters. In addition, various types of CF (including types of sizing agents) respond differently to plasma treatment. The polymer binder is of relevance as well when the deformation response of laminates is examined. This study aims to examine non-widespread techniques like low-temperature plasma treatment (with atmospheric discharge with runaway electrons, DRE) for the improvement of the mechanical properties of PPS/CF laminates, with accurate numerical estimation of the role of interlayer adhesion.

This study aims at establishing the role of the preliminary cold plasma treatment of CFs [48] and determining the optimal parameters (primarily, the process duration) of interlayer adhesion in PPS-based laminates. In this case, the investigated parameter was assessed empirically through both ILSS testing of the PPS/CF laminate samples and ILSS computer simulation implementing the finite element method (FEM). So, some conditions were varied at the PPS/CF interface. The paper is composed as follows. Section 2 describes both the studied materials and experimental methods, as well as the computer simulation technique. The results of the ILSS tests, X-ray photoelectron spectroscopy (XPS), structural examinations (SEM) and numerical simulation (FEM) are presented in Section 3, divided into corresponding Section 3.1, Section 3.2, Section 3.3 and Section 3.4. The conclusions are preceded by Section 4, which contains a discussion of the obtained results.

## 2. Materials and Methods

### 2.1. Experimental Studies

‘Fortron grade 0205B4PPS’ powder (Ticona, Sulzbach, Germany) was used as a thermoplastic binder. For reinforcement, ‘12K-300-230′ unidirectional CF tapes (UMATEX, Moscow, Russia) with a surface density of 230 g/m^2^ and a tensile strength of >4.9 GPa were loaded.

Initially, the surfaces of CFs were modified in an installation for processing materials with low-temperature plasma with atmospheric discharge with runaway electrons (DRE) [49]. The procedure was carried out under normal conditions in continuous pulse generation mode with the following characteristics: an output voltage pulse amplitude of 56 kV, a pulse rise duration of 10 ns, and a pulse half-maximum duration of 40 ns. This mode was chosen based on previous experience in processing various fibrous materials [50]. The only variable parameter was the plasma treatment duration, which was 5, 10, 15 and 20 min for every side of the CF tapes. They were placed in one layer on a conveyor and processed sequentially on each side for the preset durations by switching the movement direction left/right. In doing so, the treated material was constantly in the interelectrode zone.

After the plasma treatment, both the CF tapes and the PPS powder (after drying at *T* = 100 °C for 4 h) were stacked layer-by-layer into a mold. In the PPS/CF laminates, the component ratio of CFs 48 wt.%/PPS 52 wt.%. After placing in the mold, the laminate samples were formed by hot-pressing them with a ‘GT-7014-A’ thermo-hydraulic press (GOTECH Testing Machines Inc., Taichung, Taiwan) at a specific pressure of 6.5 MPa and a temperature of 340 °C. The cooling rate was 2 °C/min. From the resulting laminate tiles, samples of the required sizes (*h* = 2.5 mm, *b* = 5 mm, *l* = 15 mm, *L* = 10 mm) were cut with a vertical milling CNC machine for the ILSS tests according to ASTM D2344 [51]. For this purpose, an electromechanical ‘Instron 5582′ testing machine (Instron, Norwood, MA, USA) was used at a cross-head speed of 1 mm/min.

XPS analysis was performed using an ‘XPS NEXSA’ spectrometer (Thermo Fisher Scientific Inc., Waltham, MA, USA) with a monochromated Al K-Alpha X-ray source at a binding energy of 1486.6 eV. Full spectra were analyzed at a pass energy of 200 (eV) and an energy resolution of 1 eV. For obtaining high-resolution spectra, the pass energy was 50 eV, while the energy resolution was 0.1 eV. The analyzed area was 400 µm^2^. A flood gun was used to compensate for the charge.

The structure of the PPS/CF laminates was examined using a ‘Quanta 200 3D’ scanning electron microscope (FEI, Hillsboro, OR, USA) equipped with a focused electron beam at an accelerating voltage of 20 kV. For ensuring the electrical conductivity of the samples, a copper film with a thickness of about 10 nm was deposited onto the fracture surfaces using a ‘JEOL JEE-420′ vacuum evaporation installation (JEOL USA Inc., Peabody, MA, USA).

### 2.2. The Computer Simulation Technique

**Figure 1:**
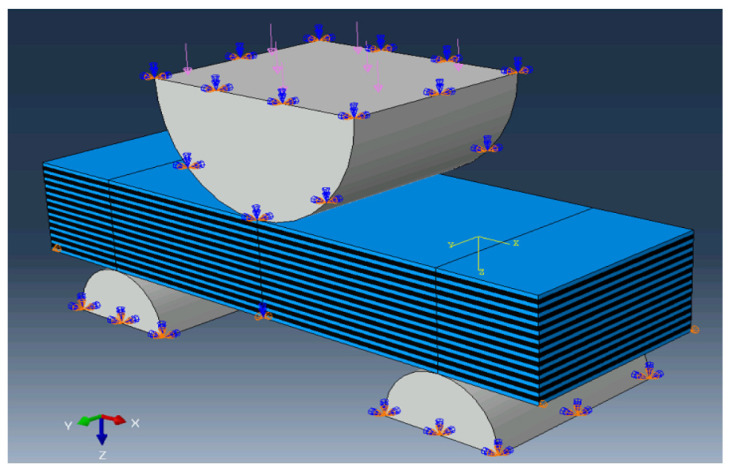
A computational domain diagram for computer simulation of three-point bending (pink arrows indicate the direction of loading; blue—rotation restrictions; orange—displacement restrictions).

The effect of interfacial adhesion upon the deformation behavior of the PPS/CF laminate samples in three-point bending was investigated theoretically using the ‘ABAQUS’ 2019 FEM-based software package (Dassault Systemes, Vélizy-Villacoublay, France). Both the sample type and the loading scheme were consistent with the experimental studies (Figure 1). The sample (15 mm long and 5 mm wide) consisted of 25 layers, each 0.1 mm thick. The PPS layers alternated with the CF ones in the *Oz* axis direction. The support span was 10 mm and the supports radius was 1.5 mm, while the loading member radius was 3 mm.

To determine the stress–strain parameters of the PPS/CF laminate samples, the contact problem of elasticity theory was solved in a 3D statement. The ‘Abaqus/Standard’ solver was implemented, which enabled us to simulate both static stress–strain states (SSS) and fracture processes.

The following boundary conditions were preset:

- The supports were rigidly fixed;
- A load was applied to the upper roller along the *Oz* axis direction, but its displacement was prohibited along the other ones.

When developing the FEM model, C3D8R volumetric tetrahedral elements with linear approximation of displacements were used. At the points of contact of the sample with the supports and the loading member, ‘Hard contact’ conditions were specified, allowing slipping but prohibiting penetration into the material. The ‘Cohesive behavior’ and ‘Damage’ contact conditions between layers were preset, preventing their mutual penetration but accepting their delamination at the studied adhesion levels. Such conditions made it possible to set the initial stiffness K at the interfaces and the d stress level required for the delamination of layers.

The PPS properties were set as follows according to experimental data reported in [52]: an elastic modulus of 3930 MPa, a Poisson’s ratio of 0.36, a tensile strength of 97.8 MPa (along the sample’s axis) and a shear strength of 35.2 MPa.

CF-containing layers (polymer-impregnated CF tape) were taken into account without the explicit consideration of enforcement (CFs), but their properties (such as the elastic and both tensile and compressive strengths) were calculated using formulas for composites unidirectionally reinforced with CFs (E_CF_ >> E_m_). Some equations were narrowed to the rule of mixtures and, as shown previously by the authors in [53], they correlated well with the computer simulation results in the case of unidirectional fibers [54,55,56,57].

In the computer simulation, it was assumed that the behavior of the CF-containing layers impregnated with PPS was transversally isotropic; as such, they contained 80 vol.% CFs and 20 vol.% PPS in the ideal case. Accordingly, the following values were considered: the tensile elastic moduli (*E*_x_ = 170 GPa, *E_y_* = *E_z_* = 10 = 4.9 GPa), the shear moduli (*G_xy_* = 3.5 GPa, *G_xz_* = *G_yz_* = 2.2 GPa), Poisson’s ratios (*μ_xy_* = 0.23, *μ_xz_* = *μ_yz_* = 0.3), the ultimate strength along the reinforcement direction (*σ_n_* = 3500 MPa) and the ultimate shear strength (*τ* = 55 MPa). The maximum normal and shear strains were assumed to be maximum *ε_n_* = *ε_τ_* = 2%, but these values varied from 2% down to 0.5% in the calculations.

## 3. Results

### 3.1. The ILSS Test

**Figure 2:**
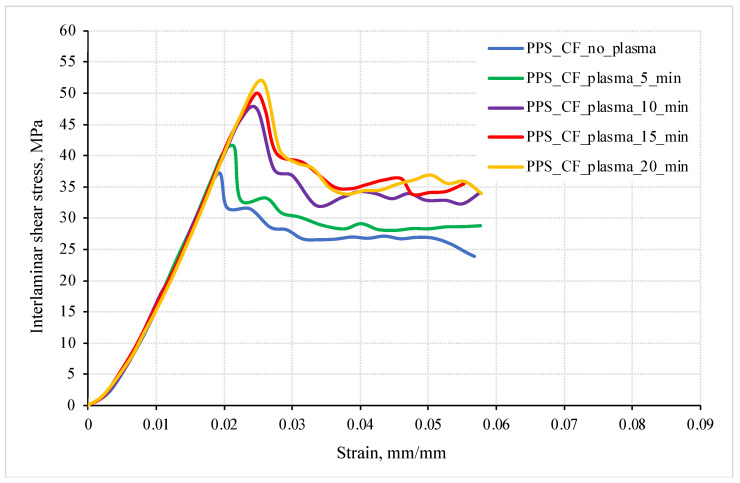
Engineering ‘strain—interlayer shear stress’ diagrams for the PPS/CF laminates.

**Figure 3:**
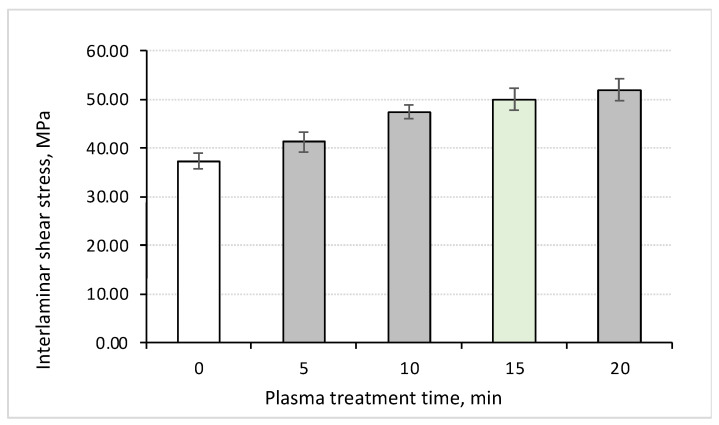
The dependences of the maximum interlayer shear stress (ILSS) on the plasma treatment duration (for 5, 10, 15 and 20 min) for the PPS/CF laminates.

It was found (Figure 2 and Figure 3) that the plasma treatment of CFs for 5 min increased the shear strength by 11%; however, a more pronounced effect (above 27%) was achieved at a duration of 10 min (Table 1). When the plasma treatment time exceeded 15 min, the shear strength values were above 50 MPa for the PPS/CF laminates (+35% compared to the sample loaded with untreated CFs). Note that these levels were nearly equal (about 52 MPa), but such a process was not rational due to both economic and technological reasons.

### 3.2. XPS Analysis

**Figure 4:**
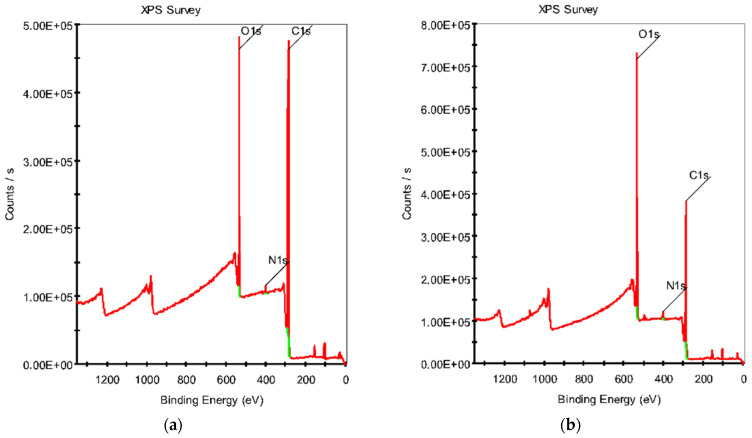

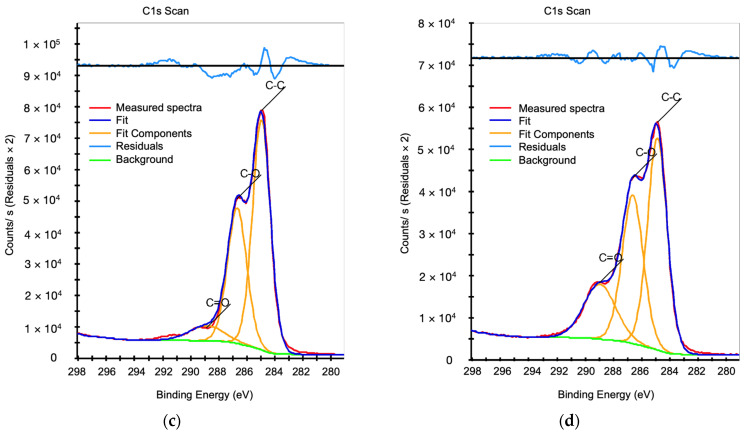
The full XPS spectra for untreated CFs (**a**) and after plasma treatment for 15 min (**b**); the high-resolution C1s spectra for untreated CFs (**c**) and after plasma treatment for 15 min (**d**).

The XPS method was proven as a highly sensitive approach to detecting changes in the chemical structure of polymers. In this study, XPS analysis was used to determine variations in the chemical composition (the C, O and N components) and concentrations of the (C–C, C–O, C=O) functional groups on the surface of the CFs before and after the plasma treatment (in order to quantitatively assess their influence). Respectively, the full spectra (Figure 4a,b) and high-resolution C1s spectra (Figure 4c,d) were registered. As a result, an increase in both oxygen concentrations (Table 2) and oxygen-containing groups (Table 3) was revealed due to prevailing surface oxidation processes, in which oxygen was absorbed from the air, and then, reacted, breaking the C–C bonds. The increase in the nitrogen content in the surface layer of CFs was explained by its presence in the air-containing plasma environment.

The high-resolution C1s spectra (Figure 4c,d) exhibited C–C (~284.8 eV), C–O (~286.5 eV), and C=O (~289 eV) components. C–N bonds had to be present at the C1s peak, but they were ignored, since the C–N components were overlapped by the C–O ones. In addition, this was caused by the wide range of possible binding energies of these components and the lower nitrogen content compared to the oxygen concentration. After the plasma treatment of CFs, a significant increase in the number of oxygen-containing groups was revealed, especially the C=O peak area, which could indicate the active formation of both carbonyl and carboxyl groups.

Thereby, the DRE plasma treatment of CFs for 15 min enabled a change in both the chemical compositions and contents of the functional groups on their surface, affecting the subsequent interaction of carbon fibers with the polymer matrix in the PPS/CF laminates. Note that polarity matching between CFs and the PPS matrix played a great role in enhancing interfacial interaction.

### 3.3. The Structural Studies

**Figure 5:**
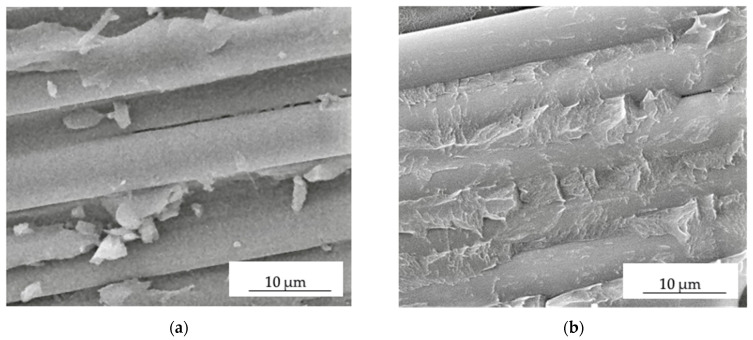
SEM micrographs of fractured PPS/CF laminates reinforced with untreated CFs (**a**) and after plasma treatment for 15 min (**b**).

SEM micrographs of the cleaved surfaces of the PPS/CF laminates after the ILSS tests are presented in Figure 5. The analysis was aimed at identifying the interaction pattern of the PPS binder with reinforcing CFs, as well as the influence of the plasma treatment on these patterns.

In the sample loaded with untreated CFs, the surface of the latter was not well wetted with the polymer (at the interlayer boundaries), while failure occurred strictly along the PPS/CF interface (Figure 5a). After the plasma treatment, CFs were well wetted with the PPS, so some adhered fragments of the fractured binder were evident on the surface of all CFs (Figure 5b). As a result, failure proceeded through a cohesive mechanism.

These results attest to the significant contribution of the plasma treatment of CFs to their adhesive interaction with the PPS matrix. The differences in structure gave rise to variations in the deformation response, namely the achievement of the maximum ILSS values for the PPS/CF laminates subjected to plasma treatment for 15 min (Figure 2).

In a previous paper [49] by the current authors, it was shown that local electrical breakdowns could occur during the DRE plasma treatment of CFs, causing damage to their surface due to the presence of a technological sizing agent. In the case of PEEK/CF laminates, this phenomenon was accompanied by a decrease in their tensile strength despite an improvement in their interlaminar shear performance.

Since the plasma treatment of CFs showed contradictory effects (eliminating the coupling agent, the local erosion of CFs, the grafting of functional groups, etc.), it was of interest to conduct an FEM-based computer simulation. In particular, the goal was to explicitly evaluate the influence of interlayer adhesion on the structure and properties of CFs (by considering layers with different characteristics). The obtained results are presented below.

### 3.4. Computer Simulation

As mentioned above, the cold plasma treatment of CFs changed their strength and adhesion to the PPS binder. As part of computer simulation, taking into account the capabilities provided by the Abaqus 2019 software package, the effect of the following factors on the strength properties of the PPS/CF laminates was assessed: (i) the *K* initial elastic stiffness at the interface (hereinafter referred to as interlayer stiffness), (ii) the d stress level required for the delamination of layers, and (iii) the *ε_τ_* shear strain in the CF-containing layers.

In three-point bending of the PPS/CF laminate samples, the *ε* strain and *σ* stress values were determined according to ASTM D 7264 [58] using the formulas

(1) $$\varepsilon =\frac{6hy}{L^{2}},$$

where *y* is the deflection of the neutral axis, *h* is the sample height and *L* is the support span, and

(2) $$\sigma =\frac{3LF}{{2bh}^{2}}$$

where *F* is the applied load and *b* is the sample width.

In three-point bending of the PPS/CF laminates after the plasma treatment of CFs, an increase in the slope of the stress–strain curves (Figure 3) can be interpreted as a change in the *K* interlayer stiffness. To assess its level, the *K* parameter was varied from 4200 to 4900 MPa, while all other conditions were equal. The obtained results are shown in Table 4.

The data presented in Table 4 enabled us to conclude that the experimentally obtained level of the *E* flexural modulus corresponded to its analytically calculated value at a *K* interlayer stiffness of 4900 MPa.

It is experimentally shown above that the strength of the PPS/CF laminates was enhanced after the plasma treatment of CFs (Figure 2 and Figure 3), which should be caused by an increase in the interlayer (interfacial) adhesion level. Therefore, its influence on the deformation behavior of the PPS/CF laminates and their fracture was analyzed.

In the framework of the formulation used in this study, it was assumed that the fracture process began when stresses and/or strains met a certain criterion (the onset of delamination) [59]. To describe the initiation and evolution of damage under the combination of normal and shear strains at the interface, it was recommended to call for effective displacement in Abaqus [60,61], which determined the peak values of the nominal deformation.

For the onset of delamination and its further evolution, the implemented criterion was the magnitude of the effective displacement at failure relative to the effective displacement at the moment of damage. Thus, this value was taken as the d interlayer adhesion level, which varied in a range from 0.002 to 0.003. These values were chosen to fall within the range obtained from the experimental data.

**Figure 6:**
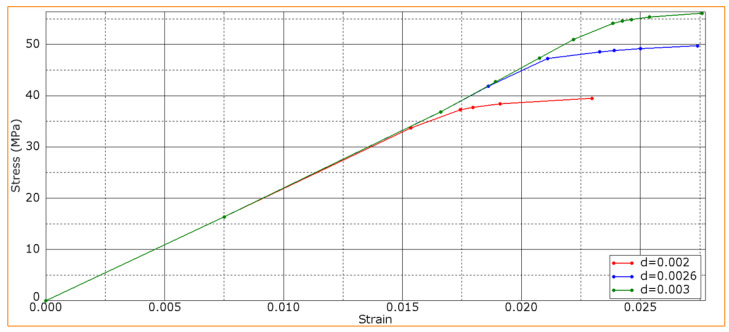
Bending strain–stress diagrams for various d interlayer adhesion levels.

Figure 6 shows the calculated bending strain–stress diagrams for the PPS/FC laminate samples at various interlayer adhesion levels. The maximum strength properties were characteristic of the sample with a d interlayer adhesion of 0.003, while the lower values were typical at *d* = 0.002. In this case, the K interlayer stiffness was constant (4900 MPa), which corresponded to the slope of the experimentally obtained curve (Figure 2).

**Figure 7:**
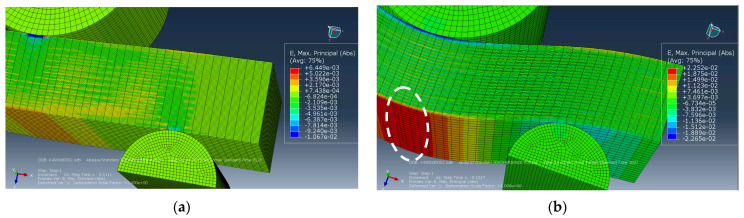
The distributions of equivalent strains: (**a**) at the onset of delamination stage and (**b**) at the crack initiation stage (*d* = 0.002).

The development of failure mechanisms was almost equal at various adhesion levels for the first loading stages. Figure 7 presents the distributions of strains at the minimum *d* interlayer adhesion level of 0.002. The onset of nonlinearity in the three-point bending strain–stress diagrams (Figure 6) was associated with the beginning of the fracture process, namely with the initiation of delamination between the upper layers of the sample under the loading member. 

According to Figure 7a, the highest tensile strains occurred in the central part of the sample in its lower CF-containing layers. The greatest strains were also evident at the center of the sample and in the PPS layers (Figure 7a). Figure 7a testifies that compressive strains were higher under the loading member in the upper part of the sample, in contrast to tensile ones in its lower part. For this reason, delamination initiated between the upper layers of the sample, located under the loading member, and then, spread to all the underlying ones.

Upon further loading, delamination developed in the lower layers, and gradually propagated from the center of the sample towards its edges. All layers were displaced relative to each other, but no delamination was found at the edges of the sample (Figure 7b). The process progressed under the action of shear stresses caused by bending of the sample beneath the loading member. However, since delamination had not yet occurred at the edges of the sample, bending developed in the areas located above the supports. At this stage, a transverse crack initiated at the center of the lower PPS layer of the sample due to the maximum tensile strains (Figure 7b). However, the nucleation of the transverse crack did not terminate the calculation, since it was associated with the local fracture of a single layer only.

**Figure 8:**
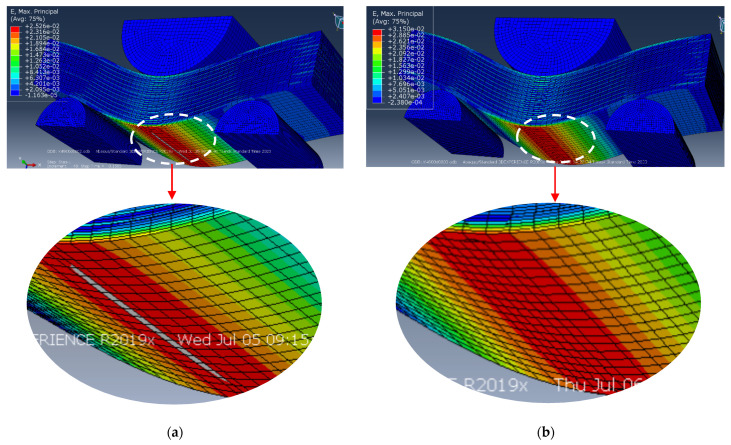
The distributions of equivalent strains at the complete delamination stage: (**a**) *d* = 0.002, (**b**) *d* = 0.003.

**Figure 9:**
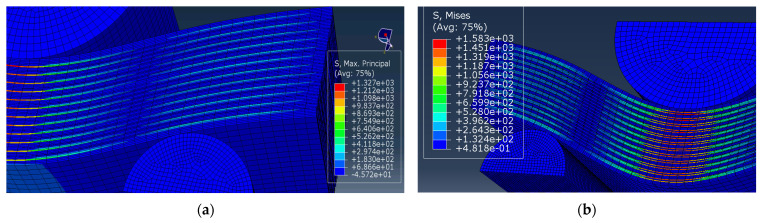
The distributions of equivalent stress at the complete delamination stage: (**a**) *d* = 0.002, (**b**) *d* = 0.003.

The distributions of stresses and strains did not fundamentally change until sample failure (according to the accepted criterion). Figure 8 and Figure 9 illustrate the distribution patterns of principal stresses and strains at various adhesion levels. It is seen that they do affect the development of the failure process. A transverse crack in the bottom layer propagated across almost the entire width of the sample (shown as an oval in Figure 8a). The maximum strain level was ~2.6% in the lower layer (Figure 8a).

At the same time, due to the large deflection magnitude at the ends of the short beam, evidence of interlayer delamination was visually distinguishable (shown by an oval in Figure 8a).

Figure 8b and Figure 9b show the distributions of strains and stresses in the PPS/CF laminate sample under three-point bending at the maximum level of interlayer adhesion, *d* = 0.003. Figure 8b and Figure 9b indicate multiple cracks initiated in the lower layers of the sample.

The distribution of equivalent stresses almost did not change prior to failure (Figure 9). Both the maximum levels of *σ* stresses and *ε* strains were higher than those at the lower adhesion level of *d* = 0.002, *σ*~1575 MPa in Figure 9b (versus *σ*~1324 MPa, Figure 9a) and *ε*~3.1% in Figure 8b (versus *ε*~2.6%, Figure 8a).

Thus, higher bending strength of the PPS/CF laminates was achieved at a greater interlayer adhesion level. This was in agreement with the experimental data (Figure 2). This is suggested to be due to the suppression of the development of shear strains at the interlayer boundaries. 

However, in addition to improving interlayer (interfacial) adhesion, the cold plasma treatment of CFs could damage their surface, reducing their tensile strength. Moreover, the longer its duration, the more their strength decreased [49]. For this reason, computer simulation was conducted when the *ε_τ_* shear strain in the CF-containing layers was varied. Both normal and shear strains were used as a criteria, whereas the interlayer adhesion level of *d* = 0.0026 was constant.

At a constant shear strain of 1%, varying normal strain from 0.5% to 2.0% did not result in a change in the strength of the PPS/CF laminates under three-point bending. The obtained dependencies completely coincided with those shown in Figure 6 for the adhesion level of *d* = 0.0026, and the sample failure exhibited a similar pattern.

**Figure 10:**
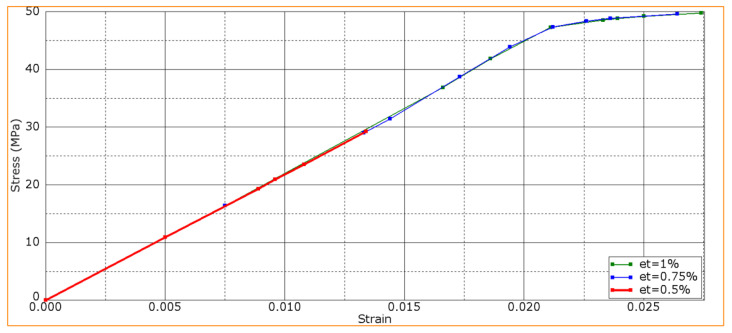
Bending strain–stress diagrams for various *ε_τ_* shear strain levels in the CF-containing layer.

At a constant normal strain of 2% and an adhesion level of *d* = 0.0026, the results of varying the *ε_τ_* shear strain in the CF-containing layers from 0.5% to 1.0% are shown in Figure 10. For *ε_τ_* = 0.75% and *ε_τ_* = 1.00%, the dependencies were almost the same. In these cases, the maximum *σ* stress was ~47 MPa in contrast to its significantly lower value of 29 MPa at *ε_τ_* = 0.5.

**Figure 11:**
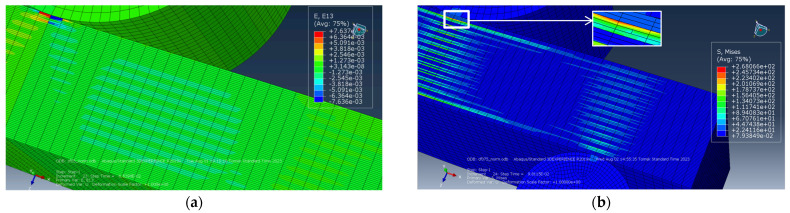
The distributions of strains (**a**) and stresses (**b**) during three-point bending at *ε_τ_* = 0.5%: shear components in the XZ plane (**a**) and Mises (**b**).

Figure 11 presents the stress distribution in the PPS/CF laminate sample under three-point bending for the minimum value of *ε_τ_* = 0.5%. In this case, a crack initiated in the upper CF-containing layers under the loading member (Figure 11a) due to the formation of a shear strain gradient (Figure 11b). The maximum shear stress did not exceed 18 MPa.

After the increase in shear strains, no cracks were evident in the CF-containing layers, but the fracture processes were different (Table 5) despite the coincidence of the curves in Figure 10:

- At the minimum *ε_τ_* value of 0.5%, a crack formed in the CF-containing layers at a *ε_b_* bending strain of ~0.8% (Figure 11), and then, the remaining layers fractured under the loading member without delamination.
- When the shear strain level increased up to 0.75%, the crack in the CF-containing layers initiated later. However, this fact generally did not affect the slope of the curve. Then, delamination initiated, causing sample failure through the progress of delamination (as shown in Figure 7, Figure 8 and Figure 9).
- At shear strain levels of ≥1%, cracks did not form in the CF-containing layers. Initially, delamination initiated, and then, the PPS matrix cracked. Sample failure proceeded through the delamination mechanism.

**Figure 12:**
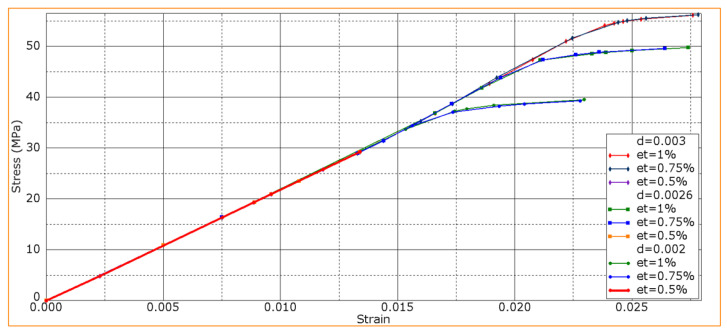
Bending strain–stress diagrams upon varying both *ε_τ_* shear strains in the CF-containing layers and *d* interlayer adhesion levels.

Figure 12 shows bending strain–stress diagrams upon varying both *ε_τ_* shear strains in the CF-containing layers and *d* interlayer adhesion levels.

According to Figure 12, varying both the ε_τ_ shear strains in the CF-containing layers and the d interlayer adhesion levels gave results similar to those for the above-described case at *d* = 0.0026. At *ε_τ_* = 0.5%, changing the d interlayer adhesion level did not affect the strength properties of the PPS/CF laminates, since the low shear strain of CFs exerted a decisive influence on the fracture process. In this study, a *ε_τ_* value of 0.5% was the critical threshold at which the strength of CFs determined this parameter for the PPS/CF laminates as a whole.

## 4. Discussion

According to the obtained results, it can be concluded that the DRE plasma treatment of CFs led to a combined physical–chemical effect: the functionalization of CFs via grafting of the active polar groups on the surface and an increase in their surface roughness [49]. This combined effect increased interlayer (interfacial) adhesion in the PPS/CF laminates by promoting chemical bonding between fibers and the polymer matrix, as well as mechanical interlocking, enhancing resistance to interlaminar cracking and the total PPS/CF contact surface area. The SEM observation clearly confirmed these justifications since the plasma-treated CFs were well wetted with the polymer. Some fragments of the fractured PPS binder were adhered to the surface of all observed CFs, and sample failure proceeded through the polymer matrix. The importance of the obtained XPS data should be noted separately, which made it possible to quantitatively characterize the plasma treatment effect.

In addition, the identified effect of ILSS improvement could be considered from the perspective of a multiscale approach to solving strength and plasticity problems [62]. In this case, the impact only on the PPS/CF interface enabled us to achieve the effective redistribution of stresses and strains. This phenomenon at the interlayer structure level could delay fracture development in the PPS/CF laminate as a whole upon the main crack initiation and propagation.

Through computer simulation while varying the adhesion level in the PPS/CF laminates, it was also clearly demonstrated that its value affected the maximum flexural strength. The visualized distributions of stresses and strains reflected that the increased interlayer adhesion level, caused by the redistribution of stresses at the interfaces, changed the pattern of the fracture process in the PPS/CF laminates and improved their bending strength. These results were fully consistent with the experimental data, which were compared with the previously reported ones on the implementation of the plasma treatment procedures in manufacturing CF-containing polymer-based laminates (Table 6).

As shown in Table 6, some progress has already been made in enhancing interlayer (interfacial) adhesion in CF-containing laminates based on both thermoreactive (epoxy) and thermoplastic (PPESK, PEEK, PPS) matrices via the plasma treatment of CFs. For this purpose, various installations have been implemented (RF, DBD, ICP, CRNOP), while the DRE plasma ones have not been widely deployed so far, although they have not provided great results. Thus, the 35% ILSS improvement for the PPS/CF laminates, achieved in this research after the DRE plasma treatment of CFs for 15 min, was significant.

In brief, the 15 min long plasma treatment resulted in an increase in the O/C ratio from 0.3 to 0.5, whereas the number of oxygen-containing groups was enlarged by 10%. The latter was significant, since according to similar XPS-based data from the literature, the average increase is equal to 5–15% [28,65,66,67,68]. It was expected by the authors that the 15 min long plasma treatment would ensure a pronounced surface activation effect (the grafting of oxygen-containing groups). However, the physical etching developed upon increasing the treatment time gave rise to damage to individual fibers. Thus, the increase in the chemical component of surface adhesion was, to a certain extent, compensated by the loss of tensile strength [49]. In the current study, this was proven by the small difference in the ILSS values for the composites treated for 15 and 20 min. As such, cold atmospheric plasma treatment for 15 min was selected as the optimal treatment based on the variety of the studied properties.

It should be noted before we state our conclusions that the authors deliberately varied only the DRE plasma treatment duration. However, the complex nature of the impact caused multidirectional changes in the chemical surface structure and the strength properties. In a forthcoming study, it is planned to vary other parameters with a constant plasma treatment duration. In addition, plasma cleaning treatment will be applied, implementing a different mode of exposure to CFs for comparison.

## 5. Conclusions

1. The plasma treatment of CFs was shown to be an effective way to activate their surface and subsequently increase their ILSS values. It was demonstrated that an acceptable ILSS level was achieved after a DRE plasma treatment duration of 15 min, whereas the effect increased slightly with a subsequent increase in duration.
2. As a result of the cold plasma treatment of CFs, their surface roughness increased and functional groups were grafted. This fact was confirmed by the XPS data, which showed a change in the chemical composition and the formation of reactive oxygen-containing groups. In turn, they made it possible to increase the adhesive bond between the polymer binder and reinforcing fibers. The SEM observation of the PPS/CF laminates clearly demonstrated a difference in adhesive interaction at the PPS/CF interface. After the DRE plasma treatment, CFs were better wetted with the polymer, and the samples cohesively fractured predominantly through the matrix, but not along the PPS/CF interface, as was observed for the sample reinforced with the untreated CFs.
3. The computer simulation results showed that raising the adhesive strength enhanced the ILSS values but reduced resistance to transverse cracking under the loading member. In general, higher flexural strength of the PPS/CF laminates was achieved with a greater interlayer adhesion level, which was consistent with the obtained experimental data.
4. The related effects of the DRE cold plasma treatment of CFs included damage to their surface and a reduction in their tensile strength. However, this fact did not affect their flexural strength under the three-point bending conditions, since CFs were fractured at the interlayer boundary earlier. Reducing the shear strain of the CF-containing layers in the normal direction (along the fibers) did not lead to its decrease for the PPS/CF laminates as a whole in three-point bending. Nevertheless, at shear strain values of <0.5%, the sample fractured through the CF-containing layers. At low shear strain of the CF-containing layers, changing the adhesion level did not affect the strength, which remained constant at the level of 29 MPa. Raising the shear strain of the CF-containing layers above 0.75% influenced the fracture process, but the strength of the PPS/CF laminates did not vary generally.

## Figures and Tables

**Table 1:** The ILSS values for the PPS/CF laminates (short-beam bending).

Plasma Treatment Duration	Ultimate Shear Strength τ, MPa	Relative Increase, %
Untreated	37.3 ± 1.6	–
*t*_plasma_ = 5 min	41.3 ± 2.1	+11
*t*_plasma_ = 10 min	47.4 ± 1.4	+27
*t*_plasma_ = 15 min	50.1 ± 2.2	+35
*t*_plasma_ = 20 min	52.0 ± 2.3	+39

**Table 2:** The chemical contents of CFs according to the full XPS spectra.

CF Types	Elemental Compositions (%)			O/C
	C1s	O1s	N1s	
Untreated	75.69	22.70	1.61	0.30
After plasma treatment for 15 min	65.50	33.25	2.03	0.51

**Table 3:** The functional groups on the surface of CFs.

CF Types	Peak Assignment (%)			Total Oxygen-Containing Groups (%)
	C–C	C–O	C=O	
Untreated	56.64	36.15	7.20	43.35
After plasma treatment for 15 min	45.70	34.87	19.43	54.30

**Table 4:** The dependence of the *E* flexural modulus on the *K* interlayer stiffness for the PPS/CF laminate samples.

Interlayer Stiffness, *K* (MPa)	Average Flexural Modulus, *E* (MPa)
The experimental results	2222
4200	2048
4500	2126
4900	2220

**Table 5:** The *ε_b_* bending strain values according to the fracture stages for various *ε_τ_* shear strains in the CF-containing layers.

The ε_τ_ Shear Strain in the CF-Containing Layers	Stage 1 (Crack Initiation in the CF-Containing Layers), *ε_b_*	Stage 2 (Initiation of Delamination), *ε_b_*	Stage 3 (the Beginning of Crack Propagation in the PPS Layers), *ε_b_*
0.50	0.0094	–	–
0.75	0.0140	0.021	–
≥1.00	–	0.021	0.026

**Table 6:** The effect of plasma pretreatment on the CF-containing polymer-based laminates.

Composite	Plasma Type	Treatment Duration, min	Relative Enhancement of ILSS/IFSS after Plasma Treatment	Reference
Epoxy/CF	Oxygen RF plasma	1	ILSS +28%	Baghery Borooj et al. [63]
Epoxy/CF	Air dielectric barrier discharge (DBD) plasma	1	ILSS +23%	Xiao et al. [40]
PPESK/CF	Inductively coupled plasma (ICP)	15	ILSS +14%	Lu et al. [64]
PEEK/CF	Cold remote (N_2_ + O_2_) plasma (CRNOP)	15	ILSS +7%	Tiwari et al. [35]
PEEK/CF	Air DRE plasma	15	ILSS +54%	Kosmachev et al. [49]
PPS/CF	Ar, N_2_ and O_2_ RF plasma	1–10 min	IFSS +150%	Yuan et al. [44]
PPS/CF	Air DBD plasma	–	IFSS +14%	Xu et al. [45]
PPS/CF	Air DBD plasma; glow discharge plasma	3 15	ILSS +23% ILSS +21%	Santos et al. [46]
PPS/CF	Air DRE plasma	15	ILSS +35%	[Current study]

## Data Availability

The data presented in this study are available on request from the corresponding author.
